# A population-based cohort study on changes in breast, lung and colorectal cancer incidence and mortality among non-Western immigrant women

**DOI:** 10.1186/s12885-023-11140-6

**Published:** 2023-07-14

**Authors:** Maarit Lamminmäki, Aku Leivonen, Sirpa Heinävaara, Mari Nygård, Giske Ursin, Suzanne Campbell, Hrefna Stefansdóttir, Elli Hirvonen, Salla Toikkanen, Ilse Merete Munk Vejborg, Sisse Helle Njor, Tytti Sarkeala

**Affiliations:** 1 Finnish Cancer Registry, Unioninkatu 22, 00130 Helsinki, Finland; 2 Data and Analytics Unit, Finnish Institute for Health and Welfare (THL), Helsinki, Finland; 3 Department of Public Health, University of Helsinki, Helsinki, Finland; 4 Department of Research, Cancer Registry of Norway, Oslo, Norway; 5 Institute of Basic Medical Sciences, University of Oslo, Oslo, Norway; 6 Department of Preventive Medicine, University of Southern California, Los Angeles, CA USA; 7 Cancer Registry of Norway, Oslo, Norway; 8 The Icelandic Cancer Society, Reykjavik, Iceland; 9 Department of Breast Examinations, Copenhagen University Hospital Herlev Gentofte, Copenhagen, Denmark; 10 University Research Clinic for Cancer Screening, Randers Regional Hospital, Randers, Denmark; 11 Department of Clinical Medicine, Aarhus University, Aarhus, Denmark

**Keywords:** Cancer incidence, Cancer mortality, Migrants, Cohort analysis, Registry data, Epidemiology

## Abstract

**Background:**

Cancer risk varies geographically, and migrants are influenced by different risk factors before, during and after migration. Increased migration from non-Western countries to the Nordic countries calls for a better understanding of the migrants’ cancer risk and the change in risk patterns over time. The aim of this study was to compare the incidence and mortality of breast, colorectal and lung cancer between non-Western immigrant and the native female population in Denmark, Finland, Iceland, and Norway.

**Material and methods:**

Data from national registries were processed and pre-analysed in each country. Multivariate Poisson regression models were used to model the relative differences in incidence and mortality as rate ratios (RR). The country-specific estimates and summary statistics were pooled together using a random effects model.

**Results:**

Non-Western immigrant women had significantly lower breast (RR 0.71, 0.65–0.78), colorectal (RR 0.72, 0.57–0.92) and lung (RR 0.55, 0.42–0.72) cancer incidence rates than native women, and the risk of these cancers among immigrant women increased with duration of residence. Differences were parallel in breast, colorectal and lung cancer mortality (RR 0.64, 0.55–0.74; RR 0.66, 0.48–0.92; RR 0.51, 0.34–0.79). Among immigrant women, higher education increased the risk for breast cancer and decreased it for lung cancer.

**Conclusion:**

The results significantly complement and add to the previous findings of cancer burden and cancer burden transition among migrants and provide evidence of a prolonged cancer risk advantage among non-Western immigrant women. However, the findings show an increasing risk of lifestyle-related cancers with increasing duration of residence in the host country. Further studies are needed to discover underlying reasons for this phenomenon.

**Supplementary Information:**

The online version contains supplementary material available at 10.1186/s12885-023-11140-6.

## Background

Breast, colorectal and lung cancer are the three most common cancers and leading causes of cancer death in women in the Nordic countries [1, 2]. Incidence of these diet and lifestyle driven cancers is lowest in non-Western regions, such as Sub-Saharan Africa and Central Asia. A transition to a Western lifestyle has, however, increased both the incidence and mortality of breast, colorectal and lung cancers also in these low-risk areas [1, 3, 4].

The number of migrant women with a non-Western background has increased considerably in the Nordic countries and elsewhere in Europe in recent decades. Whilst the overall cancer incidence and mortality among these women are still lower compared with the native female population, the risk profiles may vary depending on cancer site and the individual’s age, country of origin and destination, socioeconomic position, period of immigration and duration of residence in the host country [5–9]. Migrants moving to Western countries might experience declining risks of communicable diseases but face a high risk of chronic disease associated with adoption of unhealthy lifestyles. Vulnerability to obesity due to adoption of a more sedentary lifestyle and increased intake of energy dense foods [10] increase the risk for breast and colorectal cancer [11]. Increased migration calls for a better understanding of migrants’ cancer risk and the change in risk patterns over time.

In the Nordic countries, immigrant populations differ by periods of arrival, country of origin and marital status [12]. There is also a different share of migrant workers, students and refugees in these populations. In Finland, most immigrants have moved from the neighbouring countries, Russia and Estonia at working-age either in the 1990s or after. Labour migration in the 2000s, particularly from Poland, has influenced the structure of the immigrant population in Iceland the most. In contrast, Norway and Denmark have a longer history as host countries with established groups of immigrant workers recruited, from countries such as Turkey and Pakistan in the 1970s.

We studied the incidence and mortality of breast, colorectal and lung cancer among non-Western immigrant women and compared these to corresponding figures in the native female population in Denmark, Finland, Iceland, and Norway. Additional analyses take into account duration of residence, age at immigration, and level of education.

## Methods

### Data sources

**Fig. 1:**
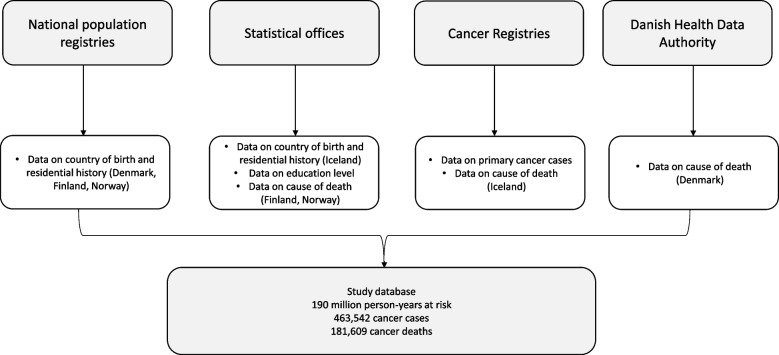
Data sources in Denmark, Finland, Iceland and Norway

We utilised the individual data in the Danish, Finnish, Icelandic and Norwegian national registries to form the study population. In each country, data on country of birth, residential history and education level were retrieved from the population registry or the statistical office; first primary cases in breast (C50), colorectal (C18–20), and lung (C33–34) cancers from the cancer registry; and cause of death from the statistical office, the health data authority, or the cancer registry. These data were linked with each other using an individual code which exists for all residents. Figure 1 shows the flow of data collection in different countries.

### Study population

Our study population consisted of all women registered as residents in 1986–2019 in Denmark, 1973–2017 in Finland, 1986–2020 in Iceland, and 1990–2015 in Norway. The country-specific follow-up times varied depending on data availability, and the beginning and coverage of registration. In each country, non-Western immigrants were defined as women born outside the Nordic countries, Western and Southern Europe, Northern America, Australia, and New Zealand. We excluded from the study population all women who emigrated within one year after immigration (2.5% of the immigrant women) and women with missing history of residence or clearly incorrect dates (0.1%) (Please see Additional file 1 for data details).

### Statistical analysis

All non-Western women were followed from the date of immigration until death, emigration, age of 95, or end of the year of the study period, whichever occurred first. The date of immigration was defined as the start of the first period of residence in the Nordic country in question, and the date of emigration as the start of the first period of residence elsewhere.

Aggregate data on the number of incident cancers, cancer deaths and person-years at risk were formed by 10-year age group and 10-year calendar period for immigrant and native women. For immigrant women, the data were also stratified by region of birth (Central and South Asia, East Asia and Pacific, Latin America and Caribbean, Middle East and North Africa, Russia and Eastern Europe, Sub-Saharan Africa; please see Additional file 1 for region details), age at immigration (0–19, 20–29, 30–39, 40 + years), duration of residence (1–9, 10–19, 20 + years), and education level at the end of follow-up (primary 0–9 years or missing, secondary 9–12 years, tertiary 12 + years).

Multivariate Poisson regression models were used to model the relative differences in incidence and mortality as rate ratios (RR) with 95% confidence intervals in the above-listed categories and subgroups. RRs were adjusted by attained age, calendar year and region of birth. First, we compared the incidence and mortality of breast, colorectal, and lung cancer between the non-Western immigrant women and the native female population. The analyses of impact of immigration age, duration of residence, and education level were performed only among migrants.

All statistical analyses were performed using the R program version 4.0.2. To ensure compliance with the EU General Data Protection Regulation (GDPR) individual data were processed and pre-analysed separately in each Nordic country, with standard R scripts developed by the Finnish Cancer Registry. After these pre-analyses, the country-specific estimates and summary statistics were sent to Finland where the estimates were pooled together using a random effects model [13].

## Results

**Fig. 2:**
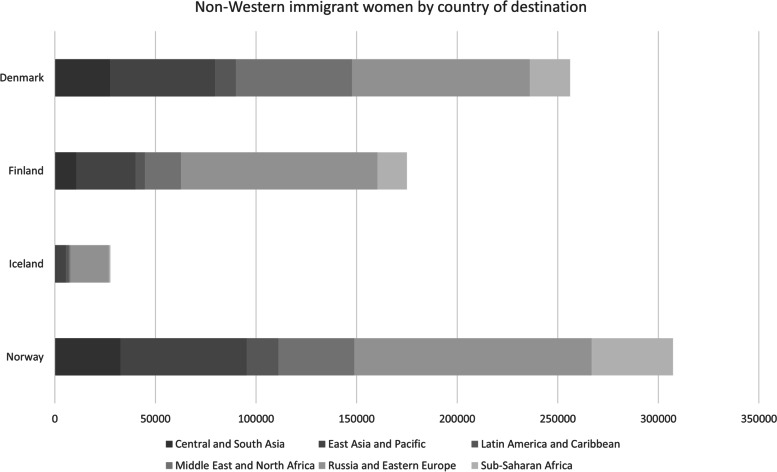
Study population, non-Western immigrant women by country of destination

There were altogether 766,033 non-Western immigrant women in the study population who contributed with 3.7% of the total of 190 million person-years at risk. Women born in Russia/the Former Soviet Union or Eastern Europe accounted for the largest group of all non-Western female immigrants (Fig. 2). However, the proportion of women migrating from the region was far larger in Finland (56%) and Iceland (69%) than in Denmark (35%) and Norway (38%), whereas the share of immigrant women from Middle East and North Africa was larger in Denmark (23%) than in the other countries (Finland 10%; Iceland 3%; Norway 12%). Furthermore, differences by individual country of birth existed within the regions, e.g., Russian population was biggest in Finland, and Polish women were in the majority in Denmark, Iceland and Norway.

Altogether there were 463,542 primary cancers in the study population (Table 1). Among the non-Western women, there were markedly more breast cancers than colorectal and lung cancers. Breast cancer was also the leading cause of cancer death.

**Table 1:** Cancer cases and deaths in the study population

	BREAST				COLORECTAL				LUNG			
	Native		Non-Western		Native		Non-Western		Native		Non-Western	
CASES												
Denmark	76 447	28%	2 306	44%	23 728	22%	503	38%	27 895	38%	364	44%
Finland	132 636	49%	1 345	25%	46 127	42%	423	32%	23 588	32%	187	23%
Iceland	4 805	2%	90	2%	1 622	1%	20	1%	1 981	3%	17	2%
Norway	59 123	22%	1 550	29%	38 032	35%	390	29%	20 107	27%	256	31%
**Total**	**273 011**	**100%**	**5 291**	**100%**	**109 509**	**100%**	**1 336**	**100%**	**73 571**	**100%**	**824**	**100%**
DEATHS												
Denmark	18 740	27%	338	39%	8 939	17%	154	33%	21 899	37%	235	41%
Finland	31 887	47%	236	27%	22 701	44%	166	35%	19 746	33%	154	27%
Iceland^a^	193	0%	< 5		104	0%	< 5		224	0%	< 5	
Norway	17 576	26%	288	33%	19 950	39%	151	32%	17 746	30%	182	32%
**Total**^a^	**68 396**	**100%**	**862**	**100%**	**51 694**	**100%**	**471**	**100%**	**59 615**	**100%**	**571**	**100%**

Number and share of breast, colorectal and lung cancer cases and deaths in the study population, among the native female population and among the non-Western women by cancer-site and country. Total number in bold text. The years of follow-up: Denmark 1986–2019, Finland 1973–2017, Iceland 1986–2020 and Norway 1990–2015

^a^Numbers under five not reported nor included in the total number due to data protection

**Fig. 3:**
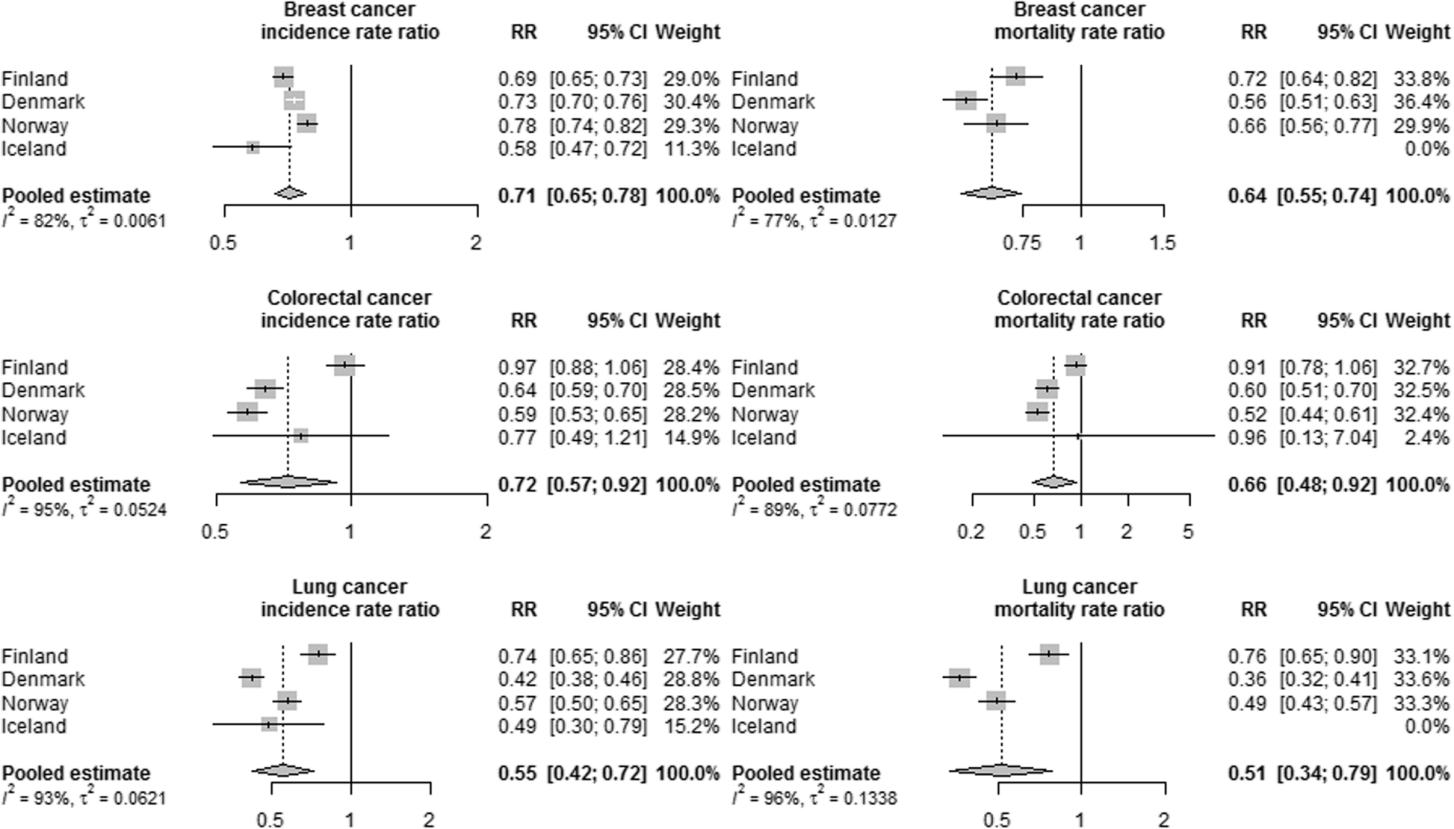
Adjusted rate ratio (RR) in cancer incidence and mortality. Breast, colorectal and lung cancer incidence and mortality among non-Western immigrant women compared to native women. (Adjusted by attained age, calendar year and region of birth. I^2^ is the heterogeneity statistic and tau^2^ is the variance of the effect size parameters across the studies)

The pooled Nordic results showed both lower incidence and lower mortality among the non-Western immigrant women compared to the native female population (Fig. 3). This was true for all studied cancers. Immigrant women had a 45% lower (RR 0.55, 0.42–0.72) lung cancer risk, a 29% lower (RR 0.71, 0.65–0.78) breast cancer risk and a 28% lower (RR 0.72, 0.57–0.92) colorectal cancer risk than native women. The differences in mortality were parallel. Between the country-specific results, the findings were most similar in breast cancer incidence. In other cancers, there were notable differences. Incidence and mortality were significantly lower among the migrants also when we looked at the pooled results by region of birth (Additional file 2). Differences in results between regions and by cancer existed.

Table 2 shows the number of cancer cases and deaths as well as the adjusted rate ratio in cancer incidence and mortality among the non-Western women by duration of residence, age at immigration and education level. The risk of studied cancers increased with duration of residence in the host country. The increase was significant in breast and lung cancers: the longer the residence, the higher the incidence and mortality. For example, non-Western women living in the Nordic countries for 20 or more years had a 56% higher breast cancer mortality compared with women who had resided in these countries 9 years or less. By immigration age, significant differences in the incidence and mortality rates were found only for breast cancer mortality. Furthermore, there were significant differences in the breast and lung cancer incidence by education level. Breast cancer incidence increased with education level. The mortality in breast cancer, on the contrary, was highest among women with fewest years of education. Both incidence and mortality in lung cancer were lowest among women with the highest education level.

**Table 2:** Cancer cases and deaths and adjusted rate ratio in cancer incidence and mortality (RR)

	BREAST				COLORECTAL				LUNG			
	Cases	RR (incidence)	Deaths	RR (mortality)	Cases	RR (incidence)	Deaths	RR (mortality)	Cases	RR (incidence)	Deaths	RR (mortality)
Duration of residence (years)												
1–9	1765	1	230	1	379	1	114	1	218	1	121	1
10–19	1627	1.05 (0.98—1.13)	264	1.25 (1.04—1.51)	439	1.23 (1.06—1.42)	152	1.29 (1.00—1.66)	216	0.91 (0.75—1.11)	156	1.14 (0.89—1.46)
20 +	1899	1.25 (1.10—1.42)	368	1.56 (1.29—1.88)	515	1.16 (0.81—1.66)	205	1.35 (0.84—2.16)	389	1.20 (1.00—1.45)	294	1.52 (1.21—1.92)
Age at immigration (years)												
40 +	1684	1	317	1	631	1	256	1	394	1	288	1
0–19	558	1.08 (0.97—1.21)	104	1.92 (1.45—2.53)	144	1.32 (0.83—2.12)	37	1.36 (0.53—3.47)	89	1.22 (0.93—1.58)	59	1.29 (0.93—1.80)
20—29	1450	1.07 (0.98—1.17)	200	1.35 (1.08—1.68)	240	0.89 (0.58—1.36)	85	1.13 (0.69—1.87)	142	1.15 (0.71—1.87)	101	1.42 (0.89—2.26)
30—39	1597	1.10 (1.01—1.20)	241	1.22 (0.86—1.73)	316	0.96 (0.76—1.21)	93	0.88 (0.63—1.22)	198	1.05 (0.82—1.33)	123	1.15 (0.86—1.54)
Education level												
Primary	2074	1	482	1	683	1	269	1	481	1	354	1
Secondary	1566	1.26 (1.18—1.35)	204	0.82 (0.69—0.98)	363	1.09 (0.96—1.25)	114	1.00 (0.79—1.26)	205	0.82 (0.64—1.06)	128	0.77 (0.57—1.05)
Tertiary	1651	1.44 (1.28—1.62)	176	0.78 (0.65—0.93)	290	0.95 (0.82—1.10)	88	0.84 (0.65—1.08)	135	0.59 (0.48—0.72)	89	0.58 (0.46—0.75)

Number of breast, colorectal and lung cancer cases and deaths and adjusted rate ratio (RR) in cancer incidence and mortality among non-Western women by duration of residence, age at immigration and education level. Pooled Nordic results. RR and 95% confidence intervals. (RRs are adjusted by attained age, calendar year and region of birth)

## Discussion

Our study is a collaborative registry study between Denmark, Finland, Iceland, and Norway, and, to our knowledge, the first analysis of cancer incidence and mortality among non-Western immigrant women combining registry data from different Nordic countries. Our results show that women born in non-Western countries have significantly lower breast, colorectal and lung cancer incidence and mortality rates than native women. Additionally, the risk of these cancers and cancer deaths among immigrant women increase with duration of residence.

Our findings are in line with existing European and Nordic studies showing differences in risk of these cancers between non-Western migrant and native women [7–9, 14–16]. The differences likely stem from lifestyle and hormonal risk factors (pregnancy history, duration of breastfeeding, hormonal contraceptive use, and hormone-replacement therapy) [10, 11, 17–19]. Smoking is the strongest lifestyle-related risk factor causing almost 90% of lung cancers and also increasing the risk of colorectal cancer [20]. Colorectal and breast cancer are also attributable to other preventable risk factors, such as alcohol intake, obesity, Western diet and physical inactivity.

Prior studies from the Nordic countries have found immigrants attending organized breast cancer screening less actively than native women [21–24]. The lower attendance rates might explain the lower breast cancer incidence as cases may remain undiagnosed in the follow-up period. However, the mortality rates are also lower among the immigrant women and could be even lower with more active screening participation. Hence, differences in breast cancer screening participation cannot explain the difference in incidence and mortality.

Immigrants may emigrate when they develop poor health or expect to die soon, but their deaths are not registered in the statistics of the country of residence. However, recent study results from Sweden do not support the overall existence of such a phenomenon, since no systematic differences were observed in health among immigrants who had emigrated compared with those who remained in the country [25].

Incidence and mortality in studied cancers differed by the immigrants’ region of birth, which may partly be explained by the differences in background cancer risk in the countries of birth [1, 26]. Our results differed also by the Nordic host countries. This, in turn, may partly be explained by the fact that incidence and mortality rates among the general female population differ in each host country [2]. For example, the age-standardised rates (/100 000) for female colorectal cancer incidence and mortality in Norway were 36.0 and 10.4 in 2015–2019, respectively, whereas in Finland they were lower, 22.2 and 6.6, respectively (please see Additional file 3 for more details). Due to different immigration patterns and age structures in the host countries, immigrant groups differ by country. This affects the composition of the study population data and might also have caused the heterogeneity in country-specific estimates.

Among the immigrant women, adaptation to new environments and exposure to risk factors may be possible explanations for the observed changes in incidence and mortality by the duration of residence. Comparing results to previous Northern European studies is, however, difficult due to the variation in the study settings. Previous studies have showed either no differences or both lower and higher rates [9, 15, 27, 28]. This may be partly due to short follow-up times used in the studies. Education level was inversely related to breast and lung cancer incidence and mortality among the non-Western women, with higher education increasing risk for breast cancer and decreasing it for lung cancer. These results are in line with earlier findings from Sweden [29] and recent Finnish cancer statistics [30]. With lung cancer in particular, the difference in incidence and mortality between education levels is highlighted. The differences observed by education level in breast cancer might also be a consequence of the higher screening attendance among women with higher levels of education. Overall, migrant women with lower education may remain at risk of social isolation and lack the knowledge of available health care services partly due to inadequate health literacy [31, 32].

Collecting data for migrant health requires consensus on data collection and similar definitions. Our study benefits from strong cultural and social connections between the Nordic countries and the existence of similar institutions – the Nordic welfare model and national population-based registries – which provide a unique basis for research combining data from different countries in the region.

A fundamental strength of our study was the combining of large individual-level data from valid and comprehensive registers from four Nordic countries, over several decades. Our study provides new information on the long-term changes in cancer incidence and mortality post-migration in the studied area. Moreover, the way in which the analyses were performed in co-operation was beneficial. Before pooling the results, the data and the variable definitions were structured, defined and verified in the same way. Uniform analyses were done systematically with the same scripts in every participating country.

Immigrant women are still relatively few and young in the countries studied here. Therefore, only the small number of cancers were observed in different subgroups, and some of the results may be sporadic. Another limitation is that we used large geographic regions including various countries and health care practices, and women with different ethnic backgrounds, which may affect the observed results. Moreover, information on potential contributing factors, such as reason for migration, marital status and origin of spouses were not available. We did not account for return migration and excluded women at their first emigration. This, together with minor registration imperfection regarding the data on residing history and causes of death, is, however, unlikely to affect the results.

## Conclusions

Registry-based longitudinal research among migrants is still scarce. This study offers evidence of a prolonged breast, colorectal and lung cancer risk advantage among non-Western immigrant women in the Nordic countries. However, our findings show that the risk of lifestyle-related cancers increased with increasing duration of residence in the host country. Further studies with different designs are needed to discover underlying reasons for this phenomenon.

## Supplementary Information

**Additional file 1:** **Supplementary table 1. **Data details by country. **Supplementary table 2. **Regions of birth (non-Western immigrants) and country groupings.

**Additional file 2:** **Supplement figure 1.** Adjusted breast cancer incidence and mortality rate ratios (RR) among non-Western immigrant women compared to native women. (Adjusted by attained age and calendar year. I² is the heterogeneity statistic and tau² is the variance of the effect size parameters across the studies). **Supplement figure 2.** Adjusted colorectal cancer incidence and mortality rate ratios (RR) among non-Western immigrant women compared to native women. (Adjusted by attained age and calendar year. I² is the heterogeneity statistic and tau² is the variance of the effect size parameters across the studies). **Supplement figure 3.** Adjusted lung cancer incidence and mortality rate ratios (RR) among non-Western immigrant women compared to native women. (Adjusted by attained age and calendar year. I² is the heterogeneity statistic and tau² is the variance of the effect size parameters across the studies).

**Additional file 3.** Age−standardized (W) cancer incidence and mortality per 100 000 among female populations in Denmark, Finland, iceland and Norway in 2015–2019.

## Data Availability

Country-specific summary data are available from the corresponding author upon reasonable request. Due to data protection regulations, the register data are not openly shared.

## Abbreviations

CI: Confidence interval
GDPR: EU General Data Protection Regulation
NCU: Nordic Cancer Union
RR: Rate ratio
