# Supplementary material for: A population-based cohort study on changes in breast, lung and colorectal cancer incidence and mortality among non-Western immigrant women
Source: BMC Cancer. 2023 Jul 14;23:665. doi: 10.1186/s12885-023-11140-6 (PMC10349457; doi:10.1186/s12885-023-11140-6)
Supplement: Supplementary file 1 — Additional file 1: Supplementary table 1. Data details by country. Supplementary table 2. Regions of birth (non-Western immigrants) and country groupings. [file 12885_2023_11140_MOESM1_ESM.docx]

Additional file 1

**Supplementary table 1.** Data details by country

| **Denmark**  **1986–2019** | Individual level data on all female residents  All women born 1933-1996 |
| --- | --- |
| **Finland**  **1973–2017** | Individual level data on female immigrants  Aggregate data on general female population |
| **Iceland**  **1986–2020** | Individual level data on all female residents |
| **Norway**  **1990–2015** | Individual level data on all female residents |

**Supplementary table 2.** Regions of birth (non-Western immigrants) and country groupings

| **Central and**  **South Asia** | Afghanistan, Armenia, Azerbaijan, Bangladesh, Bhutan, Ceylon, Georgia, India, Kazakhstan, Kyrgyz Republic, Maldives, Nepal, Pakistan, Sri Lanka, Tajikistan, Turkmenistan, Uzbekistan |
| --- | --- |
| **East Asia and Pacific** | American Samoa, Brunei, Burma, Cambodia, China, Fiji, Hong Kong, Indonesia, Japan, Korea Dem. People’s Rep., Lao PDR, Macao, Malaysia, Mongolia, Myanmar, Papua New Guinea , Philippines Republic of Korea, Samoa, Singapore, Solomon Islands, South Vietnam, Taiwan, Thailand, Vietnam |
| **Latin America and Caribbean** | Antigua and Barbuda, Antilles, Argentina, Aruba, Bahamas, Barbados, Belize, Bolivia, Brazil, Chile, Colombia, Costa Rica, Cuba, Curacao, Dominican Republic, Ecuador, El Salvador, Grenada, Guatemala, Guyana, Haiti, Honduras, Jamaica, Mexico, Nicaragua, Panama, Paraguay, Peru, Puerto Rico, St. Kitts and Nevis, St. Lucia, St. Martin, Suriname, Trinidad and Tobago, Turks and Caicos Islands, Uruguay, Venezuela, |
| **Middle East and North Africa** | Algeria, Bahrain, Egypt, Iran, Iraq, Israel, Jordan, Kuwait, Lebanon, Libya, Morocco, Oman, Palestine, Qatar, Saudi Arabia, South Yemen, Syria, Tunisia, Turkey, United Arab Emirates, Yemen |
| **Russia and**  **Eastern Europe** | Albania, Belarus, Bosnia and Herzegovina, Bulgaria, Croatia, Czech Republic, Czechoslovakia, Estonia, Hungary, Kosovo, Latvia, Lithuania, Moldova, Montenegro, North Macedonia, Poland, Romania, Russia, Serbia, Slovak Republic, Slovenia, Soviet Union, Ukraine, Yugoslavia |
| **Sub-Saharan Africa** | Angola, Benin, Botswana, Burkina Faso, Burundi, Cabo Verde, Cameroon, Central African Republic Chad , Comoros, Congo rep., Cote d'Ivoire, Djibouti, Equatorial Guinea, Eritrea, Eswatini , Ethiopia, Gabon, Gambia, Ghana, Guinea, Guinea-Bissau, Kenya, Liberia, Madagascar, Malawi, Mali, Mauritania, Mauritius, Mozambique, Namibia, Niger, Nigeria, Rhodesia, Rwanda, Senegal, Seychelles, Sierra Leone, Somalia, South Africa, South Sudan, South West Africa, Sudan, Tanzania, Togo, Uganda, Zaire, Zambia, Zimbabwe |
