# Supplementary material for: A population-based cohort study on changes in breast, lung and colorectal cancer incidence and mortality among non-Western immigrant women
Source: BMC Cancer. 2023 Jul 14;23:665. doi: 10.1186/s12885-023-11140-6 (PMC10349457; doi:10.1186/s12885-023-11140-6)
Supplement: Supplementary file 2 — Additional file 2: Supplement figure 1. Adjusted breast cancer incidence and mortality rate ratios (RR) among non-Western immigrant women compared to native women. (Adjusted by attained age and calendar year. I² is the heterogeneity statistic and tau² is the variance of the effect size parameters across the studies). Supplement figure 2. Adjusted colorectal cancer incidence and mortality rate ratios (RR) among non-Western immigrant women compared to native women. (Adjusted by attained age and calendar year. I² is the heterogeneity statistic and tau² is the variance of the effect size parameters across the studies). Supplement figure 3. Adjusted lung cancer incidence and mortality rate ratios (RR) among non-Western immigrant women compared to native women. (Adjusted by attained age and calendar year. I² is the heterogeneity statistic and tau² is the variance of the effect size parameters across the studies). [file 12885_2023_11140_MOESM2_ESM.docx]

Additional file 2

Adjusted cancer incidence and mortality rate ratios (RR) among non-Western immigrant women compared to native women by region of birth.


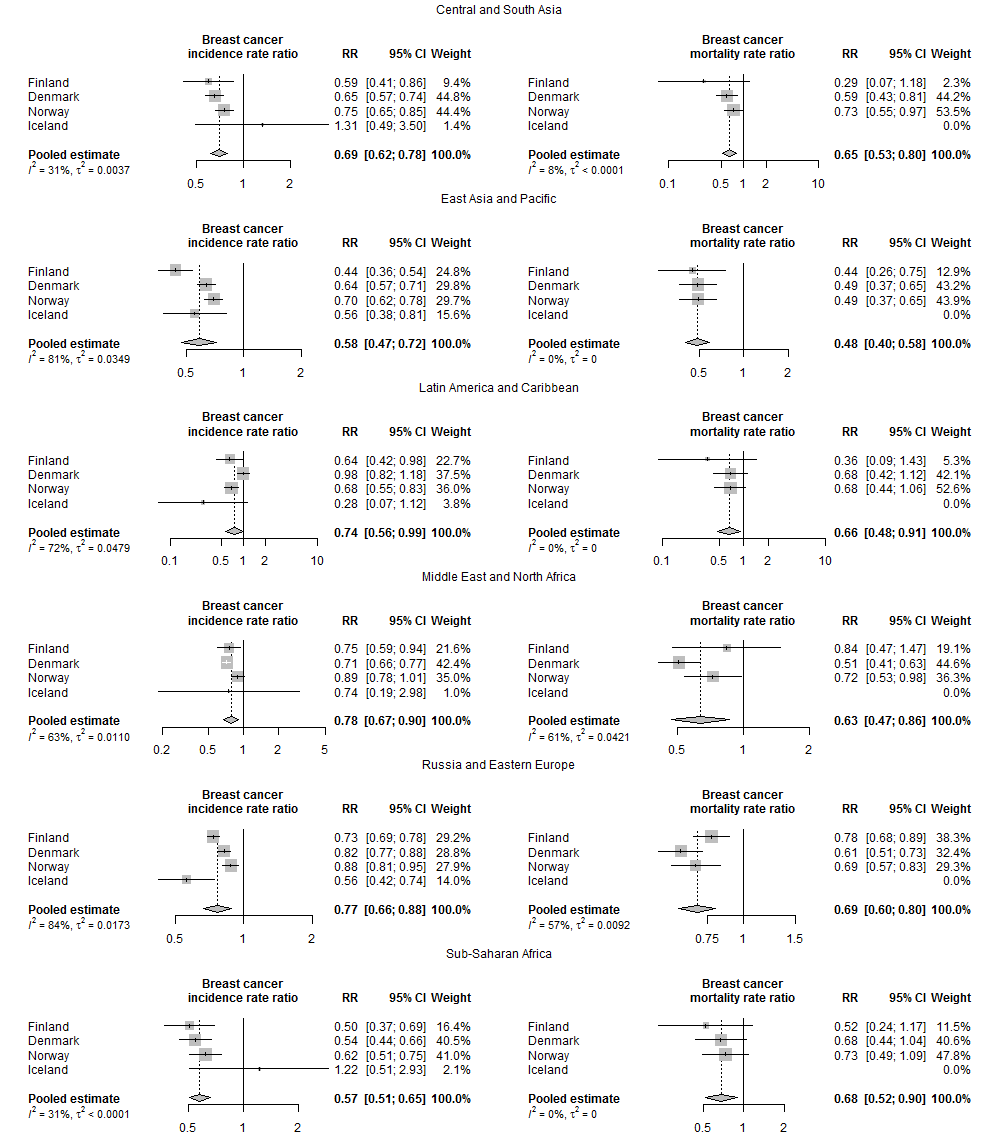


**Supplement figure 1.** Adjusted breast cancer incidence and mortality rate ratios (RR) among non-Western immigrant women compared to native women. (Adjusted by attained age and calendar year. I² is the heterogeneity statistic and tau² is the variance of the effect size parameters across the studies.)

**
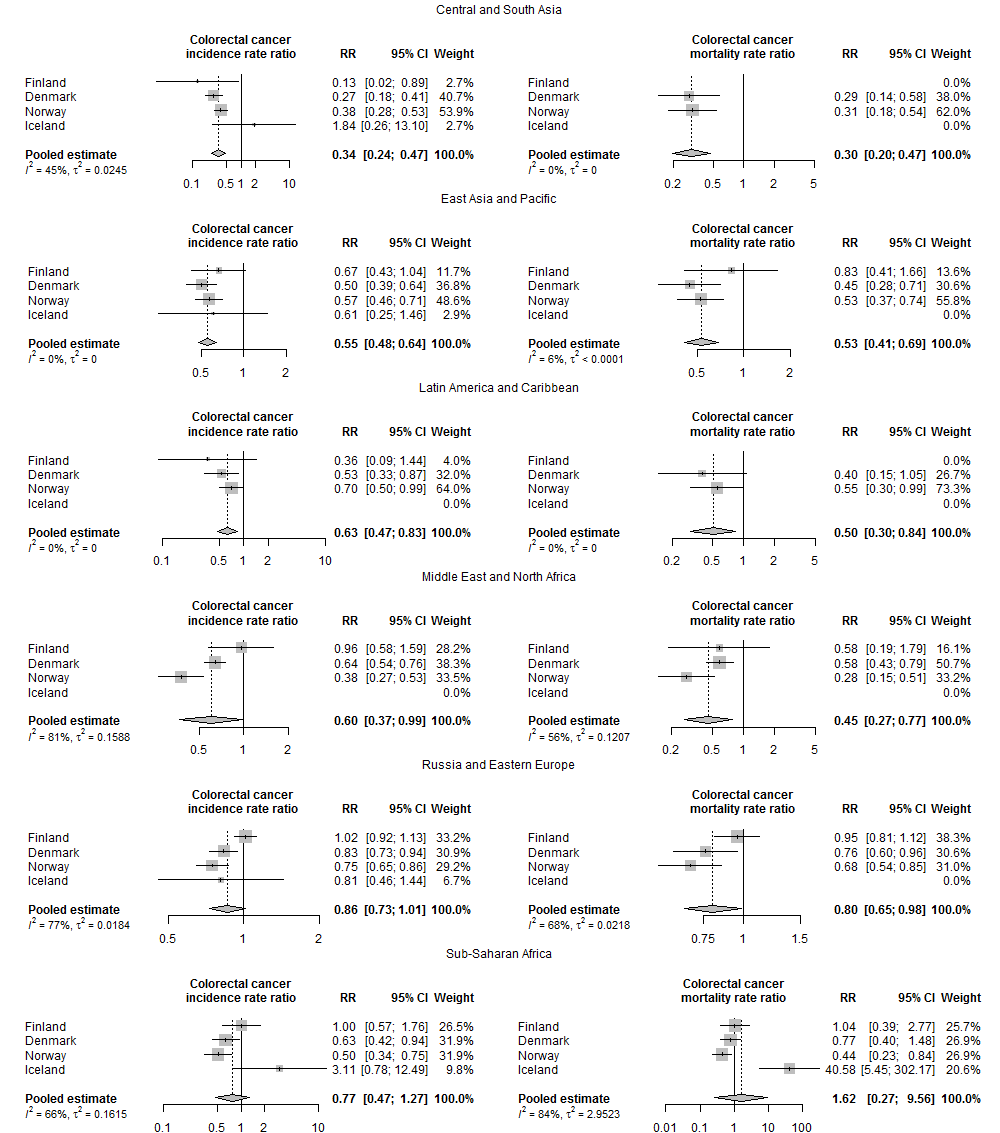
Supplement figure 2.** Adjusted colorectal cancer incidence and mortality rate ratios (RR) among non-Western immigrant women compared to native women. (Adjusted by attained age and calendar year. I² is the heterogeneity statistic and tau² is the variance of the effect size parameters across the studies.)


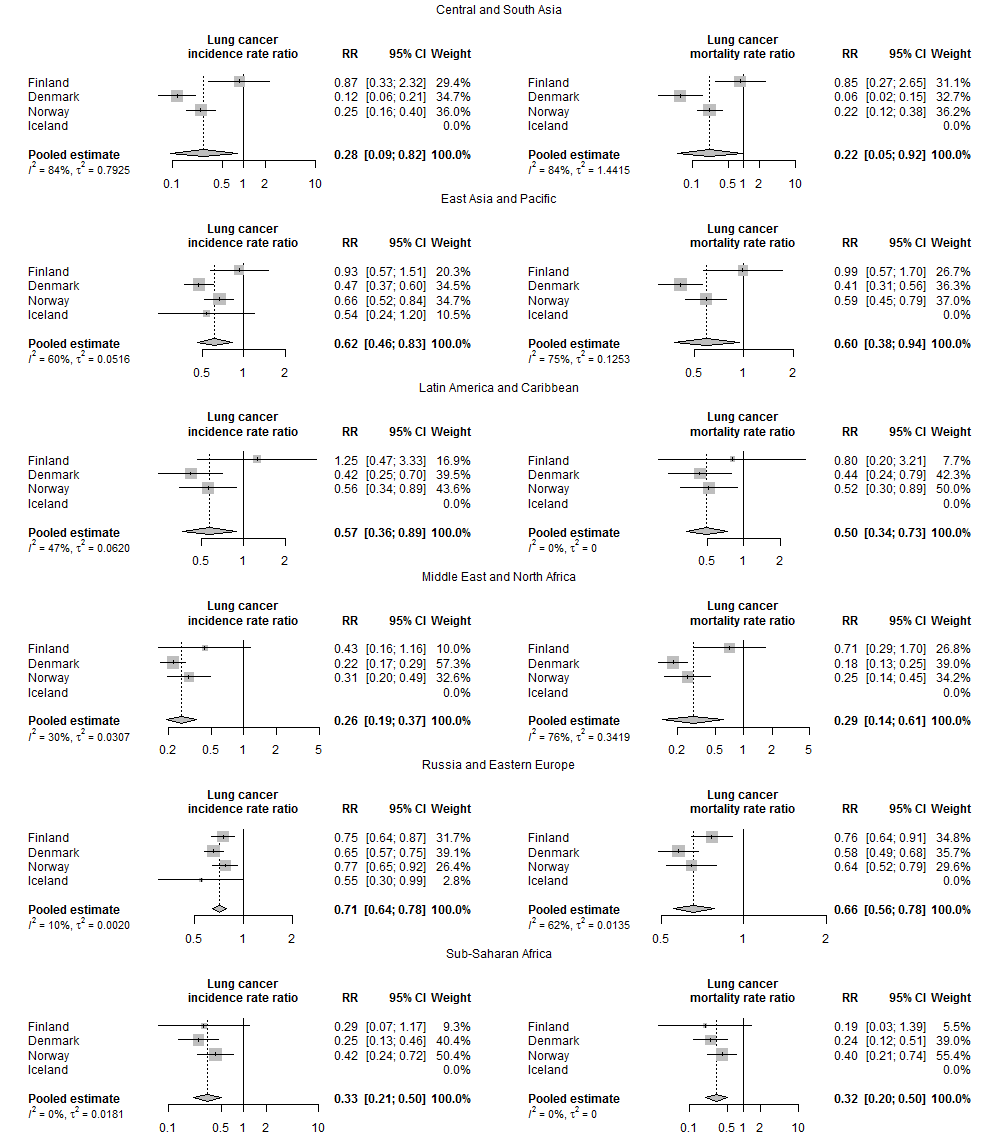


**Supplement figure 3.** Adjusted lung cancer incidence and mortality rate ratios (RR) among non-Western immigrant women compared to native women. (Adjusted by attained age and calendar year. I² is the heterogeneity statistic and tau² is the variance of the effect size parameters across the studies.)
