# Supplementary material for: A population-based cohort study on changes in breast, lung and colorectal cancer incidence and mortality among non-Western immigrant women
Source: BMC Cancer. 2023 Jul 14;23:665. doi: 10.1186/s12885-023-11140-6 (PMC10349457; doi:10.1186/s12885-023-11140-6)
Supplement: Supplementary file 3 — Additional file 3. Age−standardized (W) cancer incidence and mortality per 100 000 among female populations in Denmark, Finland, iceland and Norway in 2015–2019. [file 12885_2023_11140_MOESM3_ESM.docx]

## Additional file 3

Age−standardized (W) cancer incidence and mortality per 100 000 among female populations in 2015–2019.

|  | **BREAST** | | **COLORECTAL** | | **LUNG** | |
| --- | --- | --- | --- | --- | --- | --- |
|  | Incidence | Mortality | Incidence | Mortality | Incidence | Mortality |
| Denmark | 91.7 | 14.8 | 33.7 | 9.7 | 36.2 | 23.9 |
| Finland | 93.2 | 12.3 | 22.2 | 6.6 | 14.3 | 9.9 |
| Iceland | 89.7 | 14.9 | 27.4 | 8.3 | 29.0 | 19.7 |
| Norway | 82.2 | 10.6 | 36.0 | 10.4 | 28.0 | 16.5 |

Source: Nordcan database 2.0. Cancer statistics for the Nordic countries. <https://nordcan.iarc.fr/en/dataviz/tables>.
